# The emerging radiological features of Zika virus infection

**DOI:** 10.1590/0100-3984.2017.50.6e3

**Published:** 2017

**Authors:** Patricia Rafful, Andrea Silveira de Souza, Fernanda Tovar-Moll

**Affiliations:** 1 D'Or Institute for Research and Education (IDOR), and Federal University of Rio de Janeiro (UFRJ), Rio de Janeiro, RJ, Brazil; 2 D'Or Institute for Research and Education (IDOR), Rio de Janeiro, RJ, Brazil

In late 2015, a congenital syndrome drew worldwide attention: infection with the Zika virus (ZIKV) was associated with a marked increase in the number of cases of fetal and neonatal microcephaly or other neurodevelopmental anomalies^(1-3)^. Several epidemics of ZIKV infection occurred in tropical countries in South America (including Brazil), central Africa, Central America, and Indonesia, as well as in India^(4,5)^. A growing body of evidence allowed scientific and clinical committees to establish a causal relationship between ZIKV infection during pregnancy and adverse neurodevelopmental outcomes^(1,6-8)^. This condition has come to be known as congenital Zika syndrome (CZS).

A mosquito-borne flavivirus, transmitted by mosquitoes of the genus *Aedes*, ZIKV was first isolated in Uganda in 1947. However, vertical transmission of ZIKV was not recognized until recently. In 2016, the World Health Organization declared the ZIKV epidemic a public health emergency of international concern because of its association with microcephaly and other neurological disorders^(2,6)^. Further clinical studies demonstrated vertical and perinatal transmission, confirming the presence of ZIKV RNA in amniotic fluid and placental tissue^(2,3,9,10)^. In addition, ZIKV was isolated not only in the saliva and urine of infected adults but also in the semen of infected males, sexual transmission of the virus being therefore considered possible^(11-13)^.

Translational models using *in vitro* and *in vivo* techniques helped clarify how ZIKV infects the fetal nervous system. An *in vitro* model involving neurospheres and cerebral organoids derived from human stem cells demonstrated that human neural progenitor cells are vulnerable to ZIKV infection, showing impaired organoid growth and transcriptional dysregulation changes in the cell cycle during the proliferative phase of neurodevelopment^(3,8,14)^. It is of note that such changes have not been seen in cases of infection with similar viruses, such as the dengue virus^(14)^.

Prenatal and postnatal imaging techniques have proven to be important for mapping the spectrum of central nervous system changes in CZS. Ultrasound analysis of the anatomical structure of the brain parenchyma revealed gross neurodevelopmental changes in fetuses and neonates^(1,2)^. Computed tomography and magnetic resonance imaging have been used as adjuncts to ultrasound, confirming the morphological abnormalities but also allowing the description of subtle changes and providing a more specific evaluation of severity and prognosis of CZS^(15)^. Although there are no imaging and clinical findings that are specific for ZIKV, some have been found to be quite common and highly prevalent in a variety of cohorts of individuals infected with ZIKV. The most striking findings were microcephaly, gyral simplification (including pachygyria and agyria), hydrocephalus (with hypoplasia of the brainstem and aqueductal stenosis), linear calcifications in the cortical and subcortical white matter, cortical dysplasia, cortical development abnormalities, hypoplasia of the corpus callosum, and arthrogryposis^(13,15)^.

The study conducted by Ribeiro et al.^(16)^, published in the previous issue of **Radiologia Brasileira**, draws a very interesting trajectory for ZIKV, which is currently the most widely discussed arbovirus. The authors described the main historical aspects of the emergence and discovery of the virus, as well as how the ZIKV epidemic spread to other tropical and subtropical areas of Africa before reaching Brazil. The routes of ZIKV transmission and the associated clinical findings are well covered. The authors also detail the spectrum of neurological manifestations of ZIKV infection observed in Brazil-from the immune-inflammatory neurological involvement in adults (including meningoencephalitis, Guillain-Barré syndrome, and acute disseminated encephalomyelitis) to the dramatic, severe presentation seen in many cases of congenital malformation.

In detailing the radiological aspects of CZS, Ribeiro et al.^(16)^ describe the wide range of morphological manifestations, from the bluntest presentation of microcephaly (parenchymal calcifications and ventriculomegaly) to other findings that could also be related to the neurotropic and neurotoxic capacity of this virus. This article, together with others recently published in the literature, plays a relevant role in the dissemination of clinical, epidemiological, and radiological information regarding ZIKV infection, as well as its neurological and congenital manifestations.
